# Navigating the Diagnostic Maze: A Case Report and Narrative Review of Reversible Cerebral Vasoconstriction Syndrome

**DOI:** 10.2174/0115734056366051250604054327

**Published:** 2025-06-17

**Authors:** Xuefan Yao, Yuzhe Li, Aini He, Benke Zhao, Wei Sun, Xiao Wu, Haiqing Song

**Affiliations:** 1 Department of Neurology, Xuanwu Hospital, Capital Medicine University, Beijing, China; 2 Department of Neurology, Jixi Hospital of Traditional Chinese Medicine, Jixi, Heilongjiang, China; 3 Beijing Stroke Quality Control Center, Beijing, China

**Keywords:** Reversible cerebral vasoconstriction syndrome, Thunderclap headache, Cerebral vascular disease, Magnetic resonance angiography, Case report, Narrative review

## Abstract

**Introduction:**

Reversible cerebral vasoconstriction syndrome (RCVS) is a condition characterized by thunderclap headaches, which are sudden and severe headaches that peak within a few seconds. These headaches present diagnostic difficulties due to their diversity and low specificity, often leading to misdiagnoses and patient dissatisfaction.

**Case Presentation:**

We present the case of a 52-year-old woman with a 10-day history of recurrent thunderclap headaches. Initial imaging revealed no abnormalities, but she experienced further episodes of thunderclap headaches during hospitalization. Subsequent neurovascular imaging revealed multiple intracranial stenoses with a “string of beads” appearance, confirming the diagnosis of reversible cerebral vasoconstriction syndrome. She was treated with nimodipine, and most symptoms had resolved upon discharge, with no recurrence of headache reported during a 3-month follow-up.

**Discussion:**

Prior reviews on reversible cerebral vasoconstriction syndrome predominantly emphasized isolated symptoms or advanced neuroimaging findings, offering limited applicability in primary care services. More attention should be given to identifying clinical manifestations warranting heightened reversible cerebral vasoconstriction syndrome suspicion.

**Conclusion:**

Early recognition of reversible cerebral vasoconstriction syndrome counts in primary care services. We proposed a revised diagnostic routine that begins with clinical suspicion prompted by typical manifestations, like recurrent thunderclap headaches, female sex, and specific triggers, and recommends advanced neurovascular imaging when accessible. Extreme headache severity or deviation from prior migraine patterns should raise suspicion for reversible cerebral vasoconstriction syndrome, while diagnostic consideration should still remain in patients with transient neurological deficits, seizures, or cerebrovascular events.

## 1 INTRODUCTION

Reversible cerebral vasoconstriction syndrome (RCVS) is a rare type of cerebrovascular disease, which is also known as migrainous vasospasm, migraine angiitis, Call-Fleming syndrome, and central nervous system pseudovasculitis [1-3]. This syndrome is mostly reported among women aged 20 to 50 years, especially those receiving treatment with vasoactive drugs, such as selective serotonin reuptake inhibitors (SSRIs), serotonin-noradrenaline reuptake inhibitors (SNRIs), and triptans [4, 5]. This syndrome is characterized by recurrent thunderclap headaches (TCHs), which are similar in intensity to those caused by ruptured aneurysms but typically last for only 1~3 hours and are highly recurrent [6, 7].

The core pathogenesis of RCVS is considered to be dysregulation of cerebrovascular tone, leading to multifocal segmental vasoconstriction, which can be detected on neurovascular imaging at disease onset [8]. The vasoconstriction often presents as a “string of beads” appearance, characterized by alternating vasodilatation and centripetal propagation from distal branches of arterioles [3, 4, 9, 10]. The syndrome is often triggered by specific events, such as defecation or urination, stressful or emotional status, pregnancy or postpartum, coughing, bathing, and orgasm [3, 7, 11-13].

Although the headache is often described as “excruciating” or “insufferable,” the prognosis of RCVS is generally favorable, with most clinical headaches disappearing within 3 weeks after onset [7, 14]. However, some patients may develop potential complications, such as transient neurological deficits, seizures, posterior reversible encephalopathy syndrome (PRES), convexity subarachnoid hemorrhage (cSAH), ischemic stroke, and intracerebral hemorrhage (ICH), resulting in severe disability or even mortality [5, 14-16]. cSAH is a notable neuroimaging feature of RCVS, which is presumably caused by rupture or reperfusion injuries to the pia mater arteriole [7, 17]. The specific mechanism of RCVS is still unclear due to the difficulty of obtaining histopathological samples from the affected vessels or brain [18]. However, previous epidemiological studies have reported over 1,000 hospitalizations annually in the United States, leaving its rarity in question [19].

We presented a case of RCVS in accordance with the CARE (CAse REport) guidelines, with a detailed introduction of the patient’s journey from initial misdiagnosis to conservative drug therapy [20]. This case highlighted the importance of accurate diagnosis in seemingly ordinary headache disorders and provides valuable insights for future clinical practice.

## 2 CASE PRESENTATION

A 52-year-old female patient was admitted to our hospital on July 29^th^, 2023, with a 10-day history of recurrent, severe headaches. The patient had a history of migraines for over 30 years, but the current headache, which featured significantly more serious pain intensity, was distinct from prior occurrences. The initial episode occurred on July 19^th^, 2023, with no obvious trigger, which was characterized by an explosive, fluctuating headache accompanied by nausea but without vomiting, significant dizziness, or marked limb weakness. Despite an initial relief following the administration of ibuprofen, the patient experienced another unbearable headache the following morning during micturition, prompting emergency assessment at the local hospital. A computed tomography (CT) scan, magnetic resonance imaging (MRI), and magnetic resonance angiography (MRA) of the head revealed no significant abnormalities aside from scattered cerebral ischemic lesions, lacunar infarcts, and a right temporal pole arachnoid cyst.

A clinical diagnosis of persistent migraine was considered at the local hospital, and the patient was treated with ibuprofen and morphine for pain management, which only led to slight symptom alleviation. A lumbar puncture was further conducted on July 24^th^, 2023, revealing a cerebrospinal fluid pressure of 125 mmH_2_O, with no other notable findings. Head and neck computed tomography angiography (CTA) suggested the presence of a right internal carotid artery aneurysm at the C6 segment and moderate-to-severe stenosis in the V4 segment of the right vertebral artery. The possibility of subarachnoid hemorrhage (SAH) caused by aneurysmal rupture was considered, adding treatment with mannitol. However, the patient continued to experience intermittent and explosive headaches without significant improvement.

**Fig. (2A-C):**
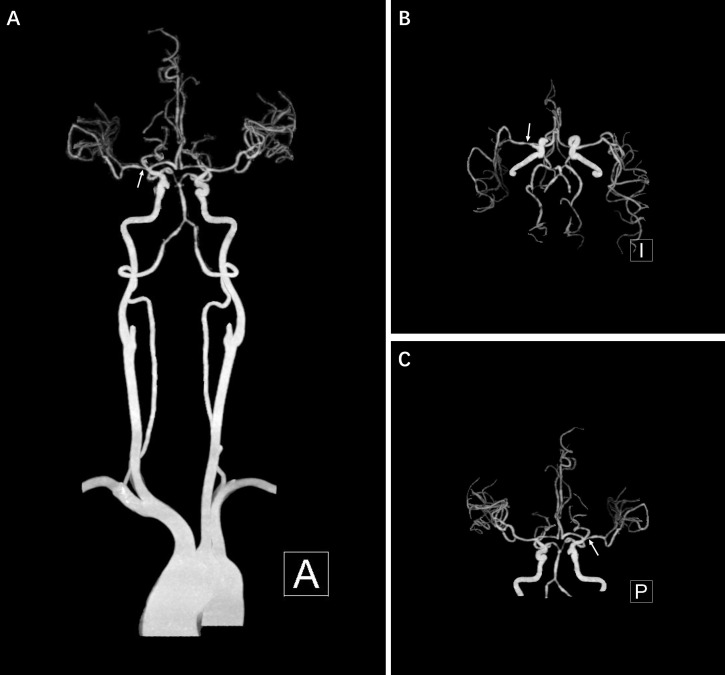
Head and neck CTA on the fourth day of admission. Severe stenosis in the M1-2 segment of the right middle cerebral artery.

For further diagnosis and treatment, the patient was transferred to our medical center on July 29^th^, 2023. The patient had been in poor spirits, with reduced appetite and insufficient sleep since onset. She often experienced headaches when urinating, which led to the placement of a catheter for drainage. Bowel movements remained generally normal. Apart from migraines, the patient had a long history of anxiety and depression. Significant abnormalities were detected in neither physical examination, neurological assessment, nor laboratory tests upon admission. Despite her migraine history, distinct manifestations in headache severity strongly suggested a novel condition. Further neurovascular imaging, including head MRI with diffusion-weighted imaging (DWI), was conducted on August 1^st^, 2023, suggesting minor hemorrhage in the right frontal-parietal subarachnoid space, raising concerns for a possible vascular malformation Fig. (**1A**-**D**). Repeated head and neck CTA on the same day revealed severe stenosis of the right middle cerebral artery and mild-to-moderate stenosis of both anterior cerebral arteries (Fig. **2**). A transesophageal echocardiogram suggested a patent foramen ovale (PFO) with positive findings following the Valsalva movement.

Considering the possibility of cerebrovascular spasm following SAH, the patient was treated with 30 mg nimodipine every 6 hours and 0.3 g extended-release ibuprofen every 12 hours. On August 3^rd^, 2023 (the sixth day after admission), she experienced transient right-sided visual impairment along with left-sided numbness and weakness, which spontaneously alleviated within 30 minutes. Subsequent MRI revealed a new infarction in the right parietal lobe Fig. (**1E**-**F**). Head MRA revealed multiple intracranial stenoses with a “string of beads” appearance Figs. (**3A**-**C**). Based on her clinical manifestations and neurovascular imaging characteristics, the patient was diagnosed with RCVS.

In light of the risk of ischemia, the treatment was adjusted to 60 mg nimodipine every 6 hours, along with intravenous injections of 120 ml butylphthalide every 12 hours and 20 ml *Ginkgo biloba* extract every day. MRA on August 8^th^, 2023 (the eleventh day after admission), suggested a fusiform dilation of the right posterior communicating artery, multiple moderate-to-severe stenoses in the left M2 segment, along with partial alleviation of intracranial stenoses Fig. (**3D**-**F**). Upon her discharge on August 9^th^, 2023, her headaches and neurological deficits had completely resolved. Considering the potential association between PFO and her neurological symptoms, the patient was evaluated for PFO closure by the cardiology department, which was postponed due to the potential risk of SAH. At the 3-month follow-up, there were no obvious symptoms of recurrence or intracranial vascular stenoses Fig. (**3G**-**I**), confirming the successful management of RCVS with conservative therapy, thus resulting in a significant decrease in her willingness to undergo PFO occlusion.

Reflecting on her winding journey of treatment, the patient expressed satisfaction with the overall outcome, noting that conservative therapy effectively alleviated the severe headaches that had troubled her for many days. She also mentioned that, given the results of conservative therapy, the PFO closure procedure was rarely considered. At the same time, she expressed some regret over the initial misdiagnoses but also showed ample understanding. She hoped that her experience could provide insights and improvements for future diagnostics and treatment of patients with RCVS.

## 3 REVIEW

While we sought to identify similar cases through prior reviews on RCVS, most works focused on isolated RCVS manifestations or advanced neuroimaging findings, offering limited applicability in primary care services. To address this gap, a narrative review was performed to summarize clinical manifestations warranting heightened RCVS suspicion, as well as its potential comorbidities between migraine, SAH, *etc*. We searched PubMed for cohort studies on RCVS and finally identified 17 associated studies with onset symptoms recorded [9, 15, 17, 21-33, 42]. A total of 2,313 participants were enrolled, with a mean age of 43.9 years old. Moreover, 76.22% of the participants were female, 67.04% had at least one trigger, and 82.33% presented TCH as one of the clinical manifestations. Details on these cohorts are presented in Table **1**.

## 4 DISCUSSION

We reported a 52-year-old female patient who, despite presenting with a seemingly common headache, had clinical characteristics and imaging features suggesting RCVS. Initially, the patient’s presentation of severe, explosive headaches could mimic a primary headache disorder, particularly given her notable history of migraines. However, the atypical clinical course indicated a divergence from the typical patterns of migraine. The discovery of scattered cerebral ischemic lesions and lacunar infarcts on initial MRI further complicated the diagnosis, but subsequently, the “string of beads” appearance in MRA provided a key clue, shifting the diagnostic focus toward RCVS.

TCH is typically associated with SAH and can hardly be distinguished from RCVS, which is based on an abnormal pattern of vasoconstriction [34]. TCH associated with SAH is usually monophasic, with pain peaking within one second, so it is highly suspected to be RCVS when the TCH shows a tendency toward relapsing-remitting episodes [6, 35]. In this case, both non-contrast head CT and lumbar puncture were conducted to identify the potential SAH. Actually, the exclusion of SAH could be tough in some cases, as cSAH is observed in 33% of RCVS patients and is regarded as one of the characteristic imaging features of RCVS [5].

On the other hand, RCVS does share many similarities with migraine, such as clinical manifestations of severe and unilateral headaches, inducing factors of physical exertion and emotional stress, and susceptibility in middle-aged women. Therefore, it is understandable that the patient was once misdiagnosed with migraine [30]. However, migraine seldom leads to prolonged intracranial vasoconstriction or severe clinical manifestations and can be relieved by ergotamine, which is, on the contrary, forbidden due to the possibility of inducing RCVS [8]. When headaches present extreme severity or deviation from prior migraine patterns, particularly when featured by thunderclap headaches, diagnosis on RCVS suspicion should be prompted.

Considering the factors above, it is quite difficult to obtain a timely and accurate diagnosis of RCVS in clinical practice, especially in primary care services where disease course and neurovascular imaging data are incomplete. Given that misdiagnosis or missed diagnosis of RCVS might lead to permanent neurological deficits, early suspicion is of considerable value. In this work, we proposed a revised diagnostic routine for RCVS to access early diagnosis in primary care services. Early diagnosis of RCVS should be highly suspected when presenting typical clinical manifestations, like recurrent thunderclap headaches, female sex, and specific triggers, and then be confirmed by advanced neurovascular imaging.

Upon suspicion of RCVS, the patient was started on nimodipine, a calcium channel blocker (CCB) that is the cornerstone of RCVS management. Meanwhile, the cerebral vasoconstriction often results in brain tissue ischemia. In cases where the etiology remains uncertain, providing symptomatic treatment for cerebral ischemia and infarction can be particularly crucial. Timely treatment not only effectively helps alleviate the symptoms but also plays a significant role in enhancing patient compliance and engagement with further treatment [36].

The presence of PFO is commonly observed in individuals with migraines, which might be involved with vasoactive substances, such as 5-HT, that bypass pulmonary circulation or impairment of vascular autoregulation [37-40]. Similarly, we speculate that vasoactive substances passing through the open foramen ovale may contribute to vascular dysfunction in RCVS patients. Stress reactions may cause fluctuations in the circulation of vasoactive substances, which enter the arterial circulation too quickly to be inactivated, thus causing recurrent abnormal vasoconstriction and TCH [40]. However, the reduction in the severity of headaches after PFO closure is less than 50%, the reasons for which still need to be further explored [41]. Future studies could verify the hypothesis through continuous measurements of vasoactive substances during acute RCVS attacks, which might offer a novel perspective on neurovascular interactions.

## CONCLUSION

Early recognition of RCVS in primary care services requires heightened clinical vigilance. We proposed a revised diagnostic routine, where initiate suspicion should be prompted by typical manifestations like recurrent thunderclap headaches, female sex, and specific triggers, and later pursuing advanced neurovascular imaging when accessible. Extreme headache severity or deviation from prior migraine patterns should raise suspicion for RCVS, while diagnostic consideration should still remain in patients with transient neurological deficits, seizures, or cerebrovascular events.

## AUTHORS’ CONTRIBUTIONS

It is hereby acknowledged that all authors have accepted responsibility for the manuscript's content and consented to its submission. They have meticulously reviewed all results and unanimously approved the final version of the manuscript.

## Figures and Tables

**Fig. (1):**
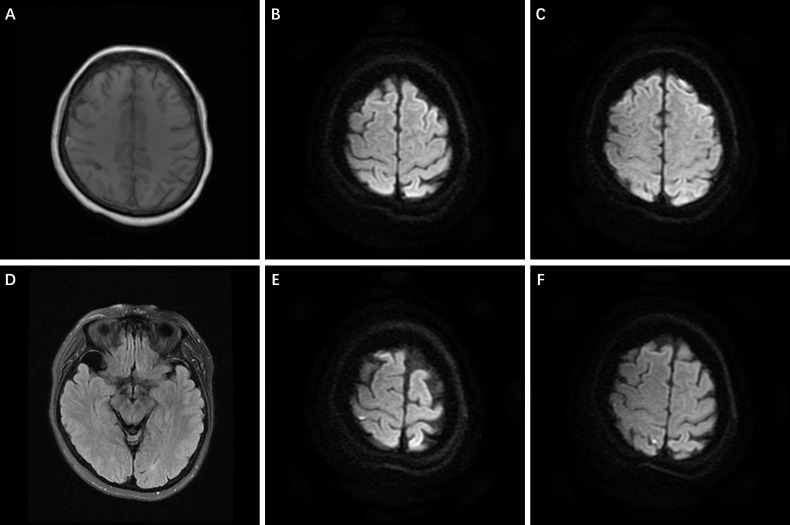
Head MRI+DWI on the fourth (**A**-**D**) and sixth (**E**, **F**) days of hospital admission. (**A**) Suspected minor SAH in the right frontal-parietal lobe, (**D**) right temporal pole arachnoid cyst, (**E**, **F**) new infarction in the cortical area of the right parietal lobe, and (**B**, **C**) comparison with the images from two days prior.

**Fig. (3):**
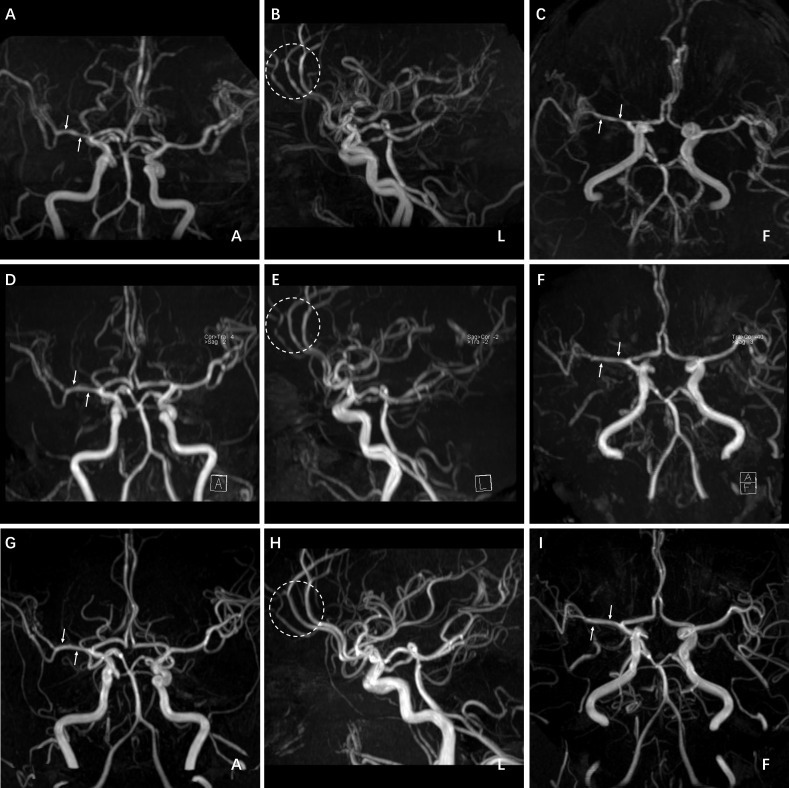
Head MRA on the sixth (**A**-**C**), eleventh (**D**-**F**) day of hospitalization, and three months after discharge (**G**-**I**). Multiple intracranial vascular stenoses presented a 'string of beads' appearance; alleviation was observed after five days and completely revolved after three months.

**Table 1:** Characteristics of cohorts on RCVS and TCH.

**Study**	**Type**	**Number**	**Mean Age**		**Female**		**Prior Migraine**		**Hypertension**		**Trigger**		**Recurrent TCH**	
Ducros 2010 [17]	Prospective	89	43.3		61 (68.54%)		24 (26.97%)		10 (11.24%)		54 (60.67%)		81 (91.01%)	
Katz 2014 [21]	Retrospective	59	47.0		52 (88.14%)		16 (27.12%)		13 (22.03%)		41 (69.49%)		-	
John 2016 [22]	Retrospective	109	44.6		86 (78.9%)		46 (42.2%)		-		58 (53.21%)		85 (77.98%)	
Lee 2017 [23]	Prospective	41	51.0		36 (87.8%)		4 (9.76%)		-		34 (82.93%)		33 (80.49%)	
Singhal 2017 [24]	Retrospective	162	44.0		126 (77.78%)		67 (41.36%)		60 (37.04%)		114 (70.37%)		120 (74.07%)	
Choi 2018 [15]	Prospective	138	51.0		113 (81.88%)		23 (16.67%)		28 (20.29%)		106 (76.81%)		105 (76.09%)	
Boysson 2018 [25]	Prospective	173	44.0		122 (70.52%)		56 (32.37%)		26 (15.03%)		99 (57.23%)		151 (87.28%)	
Shimoda 2018 [9]	Retrospective	48	40.0		35 (72.92%)		30 (62.5%)		3 (6.25%)		43 (89.58%)		45 (93.75%)	
Caria 2019 [42]	Retrospective	102	47.2		85 (83.33%)		22 (21.57%)		34 (33.33%)		57 (55.88%)		62 (60.78%)	
Cho 2019 [26]	Prospective	82	52.0		67 (81.71%)		12 (14.63%)		-		64 (78.05%)		-	
Rocha 2019 [27]	Retrospective	30	41.1		24 (80%)		8 (26.67%)		5 (16.67%)		26 (86.67%)		27 (90%)	
Boitet 2020 [28]	Prospective	171	44.1		122 (71.35%)		55 (32.16%)		26 (15.2%)		94 (54.97%)		154 (90.06%)	
Cho 2021 [29]	Retrospective	165	51.0		128 (77.58%)		29 (17.58%)		-		129 (78.18%)		148 (89.7%)	
Ling 2021 [30]	Prospective	123	46.6		86 (69.92%)		39 (31.71%)		6 (4.88%)		88 (71.54%)		-	
Patel 2021 [31]	Retrospective	799	46.3		602 (75.34%)		176 (22.03%)		414 (51.81%)		-		-	
Hathidarac 2022 [32]	Retrospective	9	46.6		8 (88.89%)		3 (33.33%)		2 (22.22%)		4 (44.44%)		-	
Strunk 2022 [33]	Retrospective	13	47.1		10 (76.92%)		1 (7.69%)		5 (38.46%)		4 (30.77%)		-	
Overall		2313	43.9		1763 (76.22%)		611 (26.42%)		632 (32.99%)		1015 (67.04%)		1011 (82.33%)	

**Table:** 

**Study**	**Transient Neurological Deficit**			**Seizure**		**PRES**		**cSAH**		**Ischemic Stroke**		**ICH**	
Ducros 2010	14 (15.73%)			4 (4.49%)		7 (7.87%)		27 (30.34%)		5 (5.62%)		30 (33.71%)	
Katz 2014	12 (20.34%)			2 (3.39%)		4 (6.78%)		15 (25.42%)		1 (1.69%)		21 (35.59%)	
John 2016	-			17 (15.6%)		-		42 (38.53%)		37 (33.94%)		18 (16.51%)	
Lee 2017	3 (7.32%)			2 (4.88%)		4 (9.76%)		2 (4.88%)		3 (7.32%)		0 (0%)	
Singhal 2017	-			23 (14.2%)		43 (26.54%)		54 (33.33%)		54 (33.33%)		21 (12.96%)	
Choi 2018	6 (4.35%)			3 (2.17%)		5 (3.62%)		7 (5.07%)		6 (4.35%)		0 (0%)	
Boysson 2018	25 (14.45%)			9 (5.2%)		13 (7.51%)		47 (27.17%)		13 (7.51%)		15 (8.67%)	
Shimoda 2018	-			1 (2.08%)		2 (4.17%)		4 (8.33%)		2 (4.17%)		1 (2.08%)	
Caria 2019	-			17 (16.67%)		31 (30.39%)		16 (15.69%)		12 (11.76%)		3 (2.94%)	
Cho 2019	-			1 (1.22%)		1 (1.22%)		3 (3.66%)		1 (1.22%)		1 (1.22%)	
Rocha 2019	-			3 (10%)		3 (10%)		14 (46.67%)		4 (13.33%)		5 (16.67%)	
Boitet 2020	28 (16.37%)			9 (5.26%)		-		47 (27.49%)		13 (7.6%)		15 (8.77%)	
Cho 2021	17 (10.3%)			1 (0.61%)		1 (0.61%)		8 (4.85%)		3 (1.82%)		1 (0.61%)	
Ling 2021	-			1 (0.81%)		2 (1.63%)		2 (1.63%)		1 (0.81%)		1 (0.81%)	
Patel 2021	-			58 (7.26%)		125 (15.64%)		287 (35.92%)		153 (19.15%)		347 (43.43%)	
Hathidarac 2022	-			2 (22.22%)		1 (11.11%)		5 (55.56%)		5 (55.56%)		0 (0%)	
Strunk 2022	-			-		2 (15.38%)		3 (23.08%)		6 (46.15%)		1 (7.69%)	
Overall	105 (12.56%)			153 (6.65%)		244 (12%)		583 (25.21%)		319 (13.79%)		480 (20.75%)	

## Data Availability

The data and supportive information are available within the article.

## LIST OF ABBREVIATIONS

CCB: Calcium channel blocker
cSAH: Convexity subarachnoid hemorrhage
CSD: Cortical spreading depression
CT: Computed tomography
CTA: Computed tomography angiography
DWI: Diffusion-weighted imaging
ICH: Intracerebral hemorrhage
MRA: Magnetic resonance angiography
MRI: Magnetic resonance imaging
PFO: Patent foramen ovale
RCVS: Reversible cerebral vasoconstriction syndrome
SNRI: Serotonin-noradrenaline reuptake inhibitor
SSRI: Selective serotonin reuptake inhibitor
TCH: Thunderclap headache

## STANDARDS OF REPORTING

CARE guidelines were followed.

## References

[1] Singhal A.B., Caviness V.S., Begleiter A.F., Mark E.J., Rordorf G., Koroshetz W.J. (2002). Cerebral vasoconstriction and stroke after use of serotonergic drugs.. Neurology.

[2] Razavi M., Bendixen B., Maley J.E., Shoaib M., Zargarian M., Razavi B., Adams H.P. (1999). CNS pseudovasculitis in a patient with pheochromocytoma.. Neurology.

[3] Calabrese L.H., Dodick D.W., Schwedt T.J., Singhal A.B. (2007). Narrative review: Reversible cerebral vasoconstriction syndromes.. Ann. Intern. Med..

[4] Chen S.P., Fuh J.L., Wang S.J., Chang F.C., Lirng J.F., Fang Y.C., Shia B.C., Wu J.C. (2010). Magnetic resonance angiography in reversible cerebral vasoconstriction syndromes.. Ann. Neurol..

[5] Singhal A.B., Hajj-Ali R.A., Topcuoglu M.A., Fok J., Bena J., Yang D., Calabrese L.H. (2011). Reversible cerebral vasoconstriction syndromes: Analysis of 139 cases.. Arch. Neurol..

[6] Perillo T., Paolella C., Perrotta G., Serino A., Caranci F., Manto A. (2022). Reversible cerebral vasoconstriction syndrome: Review of neuroimaging findings.. Radiol. Med..

[7] Ducros A. (2012). Reversible cerebral vasoconstriction syndrome.. Lancet Neurol..

[8] Singhal A.B. (2021). Posterior reversible encephalopathy syndrome and reversible cerebral vasoconstriction syndrome as syndromes of cerebrovascular dysregulation.. Continuum.

[9] Shimoda M., Oda S., Shigematsu H., Hoshikawa K., Imai M., Komatsu F., Hirayama A., Osada T. (2018). Clinical significance of centripetal propagation of vasoconstriction in patients with reversible cerebral vasoconstriction syndrome: A retrospective case-control study.. Cephalalgia.

[10] Burton T.M., Bushnell C.D. (2019). Reversible cerebral vasoconstriction syndrome.. Stroke.

[11] Wang S-J., Fuh J-L., Wu Z-A., Chen S-P., Lirng J-F. (2008). Bath-related thunderclap headache: A study of 21 consecutive patients.. Cephalalgia.

[12] Hu C.M., Lin Y.J., Fan Y.K., Chen S.P., Lai T.H. (2010). Isolated thunderclap headache during sex: Orgasmic headache or reversible cerebral vasoconstriction syndrome?. J. Clin. Neurosci..

[13] Song T.J., Lee K.H., Li H., Kim J.Y., Chang K., Kim S.H., Han K.H., Kim B.Y., Kronbichler A., Ducros A., Koyanagi A., Jacob L., Kim M.S., Yon D.K., Lee S.W., Yang J.M., Hong S.H., Ghayda R.A., Kang J.W., Shin J.I., Smith L. (2021). Reversible cerebral vasoconstriction syndrome: A comprehensive systematic review.. Eur. Rev. Med. Pharmacol. Sci..

[14] Ducros A., Boukobza M., Porcher R., Sarov M., Valade D., Bousser M.G. (2007). The clinical and radiological spectrum of reversible cerebral vasoconstriction syndrome. A prospective series of 67 patients.. Brain.

[15] Choi H.A., Lee M.J., Choi H., Chung C.S. (2018). Characteristics and demographics of reversible cerebral vasoconstriction syndrome: A large prospective series of Korean patients.. Cephalalgia.

[16] Topcuoglu M.A., Singhal A.B. (2016). Hemorrhagic reversible cerebral vasoconstriction syndrome.. Stroke.

[17] Ducros A., Fiedler U., Porcher R., Boukobza M., Stapf C., Bousser M.G. (2010). Hemorrhagic manifestations of reversible cerebral vasoconstriction syndrome: Frequency, features, and risk factors.. Stroke.

[18] Chen S.P., Wang S.J. (2022). Pathophysiology of reversible cerebral vasoconstriction syndrome.. J. Biomed. Sci..

[19] Patel S.D., Topiwala K., Otite Oliver F., Saber H., Panza G., Mui G., Liebeskind D.S., Saver J.L., Alberts M., Ducros A. (2021). Outcomes among patients with reversible cerebral vasoconstriction syndrome: A nationwide united states analysis.. Stroke.

[20] Riley D.S., Barber M.S., Kienle G.S., Aronson J.K., von Schoen-Angerer T., Tugwell P., Kiene H., Helfand M., Altman D.G., Sox H., Werthmann P.G., Moher D., Rison R.A., Shamseer L., Koch C.A., Sun G.H., Hanaway P., Sudak N.L., Kaszkin-Bettag M., Carpenter J.E., Gagnier J.J. (2017). CARE guidelines for case reports: Explanation and elaboration document.. J. Clin. Epidemiol..

[21] Katz B.S., Fugate J.E., Ameriso S.F., Pujol-Lereis V.A., Mandrekar J., Flemming K.D., Kallmes D.F., Rabinstein A.A. (2014). Clinical worsening in reversible cerebral vasoconstriction syndrome.. JAMA Neurol..

[22] John S., Singhal A.B., Calabrese L., Uchino K., Hammad T., Tepper S., Stillman M., Mills B., Thankachan T., Hajj-Ali R.A. (2016). Long-term outcomes after reversible cerebral vasoconstriction syndrome.. Cephalalgia.

[23] Lee M.J., Cha J., Choi H.A., Woo S.Y., Kim S., Wang S.J., Chung C.S. (2017). Blood–brain barrier breakdown in reversible cerebral vasoconstriction syndrome: Implications for pathophysiology and diagnosis.. Ann. Neurol..

[24] Singhal A.B., Topcuoglu M.A. (2017). Glucocorticoid-associated worsening in reversible cerebral vasoconstriction syndrome.. Neurology.

[25] de Boysson H., Parienti J.J., Mawet J., Arquizan C., Boulouis G., Burcin C., Naggara O., Zuber M., Touzé E., Aouba A., Bousser M.G., Pagnoux C., Ducros A. (2018). Primary angiitis of the CNS and reversible cerebral vasoconstriction syndrome.. Neurology.

[26] Cho S., Lee M.J., Chung C.S. (2019). Effect of nimodipine treatment on the clinical course of reversible cerebral vasoconstriction syndrome.. Front. Neurol..

[27] Rocha E.A., Topcuoglu M.A., Silva G.S., Singhal A.B. (2019). RCVS _2_ score and diagnostic approach for reversible cerebral vasoconstriction syndrome.. Neurology.

[28] Boitet R., de Gaalon S., Duflos C., Marin G., Mawet J., Burcin C., Roos C., Fiedler U., Bousser M.G., Ducros A. (2020). Long-term outcomes after reversible cerebral vasoconstriction syndrome.. Stroke.

[29] Cho S., Lee M.J., Gil Y.E., Chung C.S. (2021). RCVS–TCH score can predict reversible cerebral vasoconstriction syndrome in patients with thunderclap headache.. Sci. Rep..

[30] Ling Y.H., Wang Y.F., Lirng J.F., Fuh J.L., Wang S.J., Chen S.P. (2021). Post-reversible cerebral vasoconstriction syndrome headache.. J. Headache Pain.

[31] Patel S.D., Topiwala K., Saini V., Patel N., Pervez M., Al-Mufti F., Hassan A.E., Khandelwal P., Starke R.M. (2021). Hemorrhagic reversible cerebral vasoconstriction syndrome: A retrospective observational study.. J. Neurol..

[32] Hathidara M., Patel N.H., Flores A., Cabrera Y., Cabrera F., Koch S. (2022). Transcranial Doppler findings in reversible cerebral vasoconstriction syndrome.. J. Neuroimaging.

[33] Strunk D., Veltkamp R., Meuth S.G., Chapot R., Kraemer M. (2022). Intra-arterial application of nimodipine in reversible cerebral vasoconstriction syndrome: A neuroradiological method to help differentiate from primary central nervous system vasculitis.. Neurol. Res. Pract..

[34] Ansari S.A., Rath T.J., Gandhi D. (2011). Reversible cerebral vasoconstriction syndromes presenting with subarachnoid hemorrhage: A case series.. J. Neurointerv. Surg..

[35] Ogunlaja O.I., Cowan R. (2019). Subarachnoid Hemorrhage and Headache.. Curr. Pain Headache Rep..

[36] Pilato F., Distefano M., Calandrelli R. (2020). Posterior reversible encephalopathy syndrome and reversible cerebral vasoconstriction syndrome: Clinical and radiological considerations.. Front. Neurol..

[37] Lip P.Z.Y., Lip G.Y.H. (2014). Patent foramen ovale and migraine attacks: A systematic review.. Am. J. Med..

[38] Cao W., Shen Y., Zhong J., Chen Z., Wang N., Yang J. (2022). The patent foramen ovale and migraine: Associated mechanisms and perspectives from MRI evidence.. Brain Sci..

[39] Ashina M., Hansen J.M., Do T.P., Melo-Carrillo A., Burstein R., Moskowitz M.A. (2019). Migraine and the trigeminovascular system—40 years and counting.. Lancet Neurol..

[40] Wilmshurst P., Nightingale S. (2006). The role of cardiac and pulmonary pathology in migraine: A hypothesis.. Headache.

[41] Tobis J.M., Charles A., Silberstein S.D., Sorensen S., Maini B., Horwitz P.A., Gurley J.C. (2017). Percutaneous closure of patent foramen ovale in patients with migraine.. J. Am. Coll. Cardiol..

[42] Caria F., Zedde M., Gamba M. (2019). The clinical spectrum of reversible cerebral vasoconstriction syndrome: The Italian Project on Stroke at Young Age (IPSYS).. Cephalalgia.

